# Proportion of papillary thyroid microcarcinoma in Kerala, India, over a decade: a retrospective cohort study

**DOI:** 10.3332/ecancer.2023.1546

**Published:** 2023-05-04

**Authors:** Steve Joseph Benny, Jeffrey Mathew Boby, Ravindran Chirukandath, Togy Thomas, Ambika Vazhuthakat, Edwin Saji, Athul Raj Raju, Aju Mathew

**Affiliations:** 1 Government Medical College, Thrissur 680596, Kerala, India; 2 Government Medical College, Kozhikode 673008, Kerala, India; 3 Department of Surgery, Government Medical College, Thrissur 680596, Kerala, India; 4 Department of Pathology, Government Medical College, Thrissur 680596, Kerala, India; 5 Department of Pathology, Government Medical College, Kozhikode 673008, Kerala, India; 6 Kerala Cancer Care, Kochi, Kerala 682024, India; 7 Department of Oncology, MOSC Medical College, Ernakulam 682311, Kerala, India

**Keywords:** papillary thyroid microcarcinoma, overdiagnosis, thyroidectomy, cancer incidence, Kerala

## Abstract

**Background:**

Overdiagnosis is a phenomenon where an indolent cancer is diagnosed that otherwise would not have caused harm to the patient during their lifetime. The rising incidence of papillary thyroid cancer (PTC) in various regions of the world is attributed to overdiagnosis. In such regions, the rates of papillary thyroid microcarcinoma (PTMC) are also rising. We aimed to study whether a similar pattern of rising PTMC is found in Kerala, a state in India, where there has been a doubling of thyroid cancer incidence over a decade.

**Methods:**

We conducted a retrospective cohort study in two large government medical colleges, which are tertiary referral facilities in the state of Kerala. We collected data on the PTC diagnosis in Kozhikode and Thrissur Government Medical colleges from 2010 to 2020. We analysed our data by age, gender and tumor size.

**Results:**

The incidence of PTC at Kozhikode and Thrissur Government Medical colleges nearly doubled from 2010 to 2020. The overall proportion of PTMC in these specimens was 18.9%. The proportion of PTMC only marginally increased from 14.7 to 17.9 during the period. Of the total incidence of microcarcinomas, 64% were reported in individuals less than 45 years of age.

**Conclusion:**

The rise in the number of PTCs diagnosed in the government-run public healthcare centres in Kerala state in India is unlikely to be due to overdiagnosis since there was no disproportionate rise in rates of PTMCs. The patients that these hospitals cater to may be less likely to show healthcare-seeking behavior or ease of healthcare access which is closely associated with the problem of overdiagnosis.

## Introduction

Thyroid cancer (TC) is the most prevalent endocrine malignancy accounting for 3.3% of all the neoplasms reported worldwide in the year 2018 [1]. Papillary thyroid cancers (PTC) account for >90% of TCs, followed by follicular thyroid carcinoma (4.5%) and Hurthle cell carcinomas (1.8%). In recent decades there has been a dramatic surge in the incidence of PTC all over the world. By 2030, it will be the fourth most prevalent cancer globally [2].

Overdiagnosis is defined as the detection of either non-progressive cancers or very slow-growing cancers such that individuals die from something else before the cancer ever causes symptoms. The rise in TC diagnosis limited to early-stage indolent variety coupled with a stable mortality rate suggests an overdiagnosis of this malignancy. The rising incidence of PTC in various regions of the world is attributed to overdiagnosis. In such regions, the rates of papillary thyroid microcarcinoma (PTMC) are also rising. PTMC is a subtype of papillary thyroid tumor defined as ≤10 mm in diameter. PTMC is classified as non-incidental or incidental. Incidental PTMC is commonly diagnosed on histopathological examination following thyroid surgery for benign thyroid disease. However, some nodules ≥10 mm may also be diagnosed incidentally as they may be unpalpable depending on their location, lack of symptoms or patient factors like size or girth of neck. Non-incidental PTMC is usually diagnosed based on fine-needle aspiration biopsy and local or distant metastasis. The cancer-specific survival rate of micropapillary TC is about 100% as observed from studies [3–7].

India has one of the lowest average TC incidence rates in the world. However, the state of Kerala has an age-adjusted TC incidence rate of 13.3, ranking it eighth among the most affected regions of the world [8]. The state of Kerala has an outstanding profile in educational and health care standards compared with the other states of India and is on par with some of the high-income countries [9–12]. We aimed to study whether a similar pattern of rising PTMC is found in Kerala, like in other affluent regions of the world where the rising TC incidence is due to overdiagnosis.

## Materials and methods

We conducted a retrospective cohort study in two large government medical colleges in Kerala – Kozhikode and Thrissur. These institutions are considered tertiary referral facilities in the public sector. We included patients who were diagnosed with PTC between January 2010 and December 2020. All patients must have undergone a type of thyroid surgery. Institutional ethics approvals were obtained from both medical colleges.

Data on patient demographics and histopathological features of PTC were collected from medical records. Data were then analysed to determine the proportion of PTMC among the PTCs diagnosed during the study period. We analysed our results by categories of age at diagnosis, gender and size of the tumor.

### Statistical analysis

Descriptive analysis was used for summary statistical analyses. Data on patient demographics and clinicopathological features were reported as absolute numbers and as well as percentages.

## Results

1,805 patients with PTCs were diagnosed during the study period (1,422 females and 383 males). The incidence of PTMC was 18.9%.

### Proportion of PTMC in different age groups

**Figure 1.**
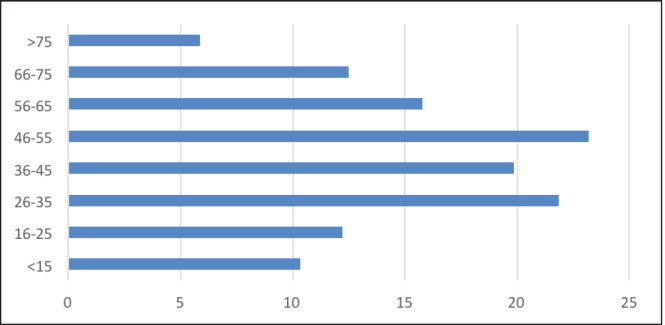
Proportion of PTMCs by age.

When analysed by 10-year age groups, most of the patients with PTC were in the age group of 36–45 (27.1%) (Figure 1). However, the highest proportion of PTMC (23.2%) was noted in the age group of 46–55. 63.9% were reported in individuals less than 45 years of age.

### Proportion of PTMC in males compared to females

**Figure 2.**
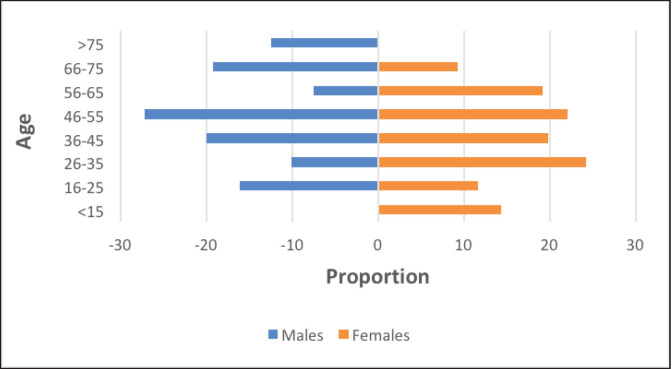
Age distribution of PTMC.

The incidence of PTMC in men (16.9%) was slightly lower than in women (19.4%) (Figure 2). Also, while the peak incidence age group in men corresponded to that seen overall (46–55 years); among women, the peak incidence of PTMC was found to be in a younger age group of 26–35.

### Size of the tumor

**Figure 3.**
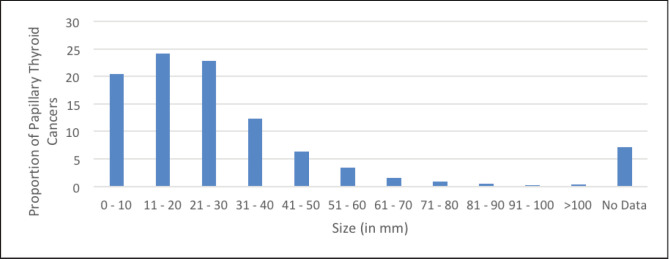
Proportion of PTCs by tumour size.

The highest proportion of PTCs was in the category of 11–20 mm (24.13%) followed by 21–30 mm (22.76%) and ≤10 mm (20.37%) (Figure 3). Excluding those tumors where size was not mentioned, the incidence of PTMC was 18.9% in this study cohort. 45% of all PTC in the study was below palpable size (2 cm).

### Numbers of PTMCs and PTCs and their proportion throughout the study

**Figure 4.**
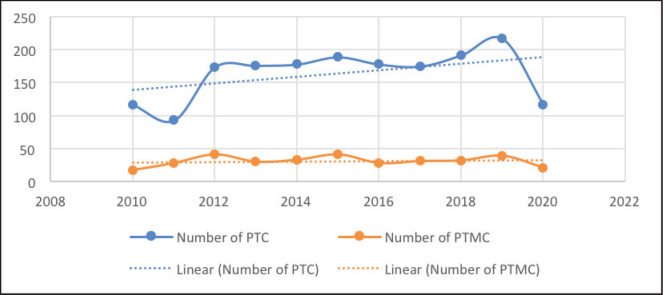
Number of PTC and PTMCs over the study period.

**Figure 5.**
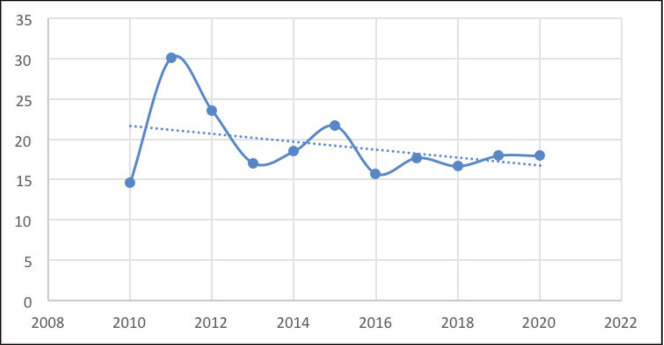
Proportion of PTMCs over the study period.

The number of PTCs decreased slightly from 116 in 2010 to 93 in 2012 but then rose sharply to 174 in 2012. The number of PTCs then gradually rose to 217 over the next 7 years (Figure 4). However, in 2020, far fewer PTCs were diagnosed, likely due to COVID19. The number of PTCs showed a moderate upward trend over the decade (Figure 4). On the other hand, the number of PTMCs saw wide fluctuations with minimal absolute change during the period of the study. Consequently, the proportion of PTMCs demonstrated a moderate downward trend during the study period (Figure 5).

## Discussion

Previous studies [1, 13] suggested the following points as supporting TC overdiagnosis: (i) substantial increase in incidence but with a large variability among and within countries; (ii) the mortality trends remained stable; (iii) the increase involved mostly small papillary subtypes; (iv) young or middle-age adults mostly affected. The variability of incidence of TC in the different states combined with stable mortality rates has well been demonstrated in other similar studies done in the region [1, 8]. In this study, we aimed to find out whether the increase in the diagnosis of PTC was closely associated with a rise in PTMCs.

Although we found a significant rise in the incidence of PTCs, we did not find a proportionate rise in PTMCs in the centres. Mathai *et al* [14] performed a study to assess the rising trend of papillary microcarcinomas in a single institution in the Thiruvananthapuram district of Kerala on a population very similar to ours, albeit in a private healthcare setting. They found the frequency of microcarcinomas in their studies to be 20.9% which was very similar to the 18.9% seen in our study. Other studies by Lam *et al* [15], Kaliszewski *et al* [16] and Girardi *et al* [17] found higher frequencies of microcarcinomas at 27.8%, 39.7% and 42.1%, respectively. However, John *et al* [18] and Gürleyik *et al* [19] observed a lower incidence of 7.2% and 9.4% in their studies of thyroidectomy specimens.

In their study, Mathai *et al* [14] observed an increase in PTC incidence accompanied by a significant increase in the frequency of microcarcinomas (they do not provide the data to support their assertion). Kaliszweski *et al* [16] and Jung *et al* [20] also reported a significant increase in the proportion of PTMC over the years. Abboud *et al* [21] found that the frequency of microcarcinomas remained stable for 11 years and that the increase in PTC was independent of microcarcinomas. Vlassopoulou *et al* [22] in a 30-year study also observed a stable frequency of microcarcinoma.

Interestingly, while the absolute numbers of both PTC and PTMC decreased in 2020, presumably due to the effects of the COVID-19 pandemic, the proportion of PTMCs remained similar to that of the previous years. If overdiagnosis played a role in the rise in PTC numbers in our study, there would have been a disproportionate fall in the number of PTMCs diagnosed compared to the PTCs, especially during COVID19, where a lot of avoidable medical and surgical procedures were delayed.

Our findings deviate from the generally accepted ideas of overdiagnosis as it related to PTMCs – in regions with overdiagnosis, an increasing incidence of PTMC could also be observed. But, on further thought, it does support the hypothesis that the increase in the incidence of PTCs in Kerala state in India is from the phenomenon of overdiagnosis. Our study revealed a higher proportion of patients in the 11–20 mm and 21–30 mm size categories compared to tumors less than 10 mm perhaps as a result of the introduction of ultrasonographic guidelines in the last decade and their implementation for the management of thyroid nodules. A study conducted among Japanese patients also recommends close observation for PTMCs unless it shows features of tumor progression [23]. The rising incidence of TC has been strongly linked to increased access to a standard health care system with national screening programs for the public and in regions with higher literacy and socioeconomic levels [13, 24–26]. PTC overdiagnosis arises from greater health-seeking behavior and easier access to healthcare. Our study population does not fit such a group of individuals – our data arose from two large government-run hospitals in Kerala which serve the relatively lower socioeconomic strata of the population. These state-run institutions are bound by economic and manpower constraints, thereby maximum utilisation of limited resources is prioritised. This supports the notion that overdiagnosis is a phenomenon in affluent societies with greater access and utilisation of healthcare.

Therefore, a similar study in large private centres may be needed to test our hypothesis. But it also brings up the issue of the significant increase in the incidence of PTCs over a decade. If there is no health-seeking behavior or ease of healthcare access, without a proportional rise in PTMCs, why did PTC rates increase substantially over time? Such contradictory findings just point to the fact that the high incidence of TC may not all be because of overdiagnosis, and further research into various risk factors is emergently needed. A large prospective multicentre cohort study is also an urgent area of unmet need in Kerala.

Our study has a few limitations. First, we do not have data on patients being treated in non-governmental institutions in Kerala. Second, a detailed analysis of the histopathological samples was not available. This might have helped to rule out possible chances of misdiagnosis. Third, being a retrospective study, the lymph node status of our patients could not be analysed. And finally, we do not have the mortality data for our study population.

## Conclusion

The rise in the number of PTCs diagnosed in the centers under study from 2010 to 2020 is unlikely to be due to overdiagnosis. Although the state of Kerala experiences an overdiagnosis of TCs, it may not be as prevalent in government-run hospitals like the ones in our study that are economically constrained. The patients that our hospitals cater to may be less likely to show healthcare-seeking behavior which is a prerequisite to the problem of overdiagnosis. This supports the notion that overdiagnosis is a phenomenon in affluent societies. A similar study, conducted on people of diverse backgrounds using both public and private healthcare services would be helpful to understand the plausibility of overdiagnosis causing the profound rise in the incidence of TC in Kerala.

## Conflicts of interest

The authors declare no conflict of interest.

## Funding

This research received no external funding.

## Figures and Tables

**Table 1.** Total number of micropapillary carcinomas diagnosed among papillary thyroid carcinomas in Thrissur and Calicut medical colleges from 2010 to 2020.

Thrissur Medical College												
Year	2010	2011	2012	2013	2014	2015	2016	2017	2018	2019	2020	Total
Number of micropapillary cancers	7	14	23	19	17	27	9	11	11	14	8	160
Number of papillary cancers	58	44	96	108	103	113	64	49	58	69	29	791
Proportion of micropapillary carcinomas	12.1	31.8	24.0	17.6	16.5	23.9	14.1	22.5	19.0	20.3	27.6	20.2
Calicut Medical College												
Year	2010	2011	2012	2013	2014	2015	2016	2017	2018	2019	2020	Total
Number of micropapillary cancers	10	14	18	11	16	14	19	20	21	25	13	181
Number of papillary cancers	58	49	78	68	75	76	114	126	134	148	88	1,014
Proportion of micropapillary carcinomas	17.2	28.6	23.1	16.2	21.3	18.4	16.7	15.9	15.7	16.9	14.8	17.9
Cumulative numbers												
Year	2010	2011	2012	2013	2014	2015	2016	2017	2018	2019	2020	Total
Number of micropapillary cancers	17	28	41	30	33	41	28	31	32	39	21	341
Number of papillary cancers	116	93	174	176	178	189	178	175	192	217	117	1,805
Proportion of micropapillary carcinomas	14.7	30.1	23.6	17.1	18.5	21.7	15.7	17.7	16.7	18.0	18.0	18.9

**Table 2.** Total number of micropapillary carcinomas diagnosed among papillary thyroid carcinomas in Thrissur and Calicut medical colleges from 2010 to 2020 (females).

Thrissur Medical College												
Year	2010	2011	2012	2013	2014	2015	2016	2017	2018	2019	2020	Total
Number of micropapillary cancers (females)	5	10	20	18	15	24	7	10	8	11	5	133
Number of papillary cancers (females)	46	34	82	92	83	101	54	39	48	59	21	659
Proportion of micropapillary carcinomas (females)	10.9	29.4	24.4	19.6	18.1	23.7	13.0	25.6	16.7	18.6	23.8	20.2
Calicut Medical College												
Year	2010	2011	2012	2013	2014	2015	2016	2017	2018	2019	2020	Total
Number of micropapillary cancers (females)	6	11	15	9	12	13	15	17	16	18	11	143.0
Number of papillary cancers (females)	39	37	64	50	48	57	96	101	99	109	63	763
Proportion of micropapillary carcinomas (females)	15.3	29.8	23.4	18.0	25.0	22.8	15.6	16.8	16.2	16.5	17.5	18.8
Cumulative numbers												
Number of micropapillary cancers (females)	11	21	35	27	27	37	22	27	24	29	16	276
Number of papillary cancers (females)	85	71	146	142	131	158	150	140	147	168	84	1,422
Proportion of micropapillary carcinomas (females)	12.9	29.6	24.0	19.0	20.6	23.4	14.7	19.3	16.3	17.3	19.1	19.4

**Table 3.** Total number of micropapillary carcinomas diagnosed among papillary thyroid carcinomas in Thrissur and Calicut medical colleges from 2010 to 2020 (males).

Thrissur Medical College												
Year	2010	2011	2012	2013	2014	2015	2016	2017	2018	2019	2020	Total
Number of micropapillary cancers (males)	2	4	3	1	2	3	2	1	3	3	3	27
Number of papillary cancers (males)	12	10	14	16	20	12	10	10	10	10	8	132
Proportion of micropapillary carcinomas (males)	16.7	40	21.4	6.3	10	25	20	10	30	30	37.5	20.5
Calicut Medical College												
Year	2010	2011	2012	2013	2014	2015	2016	2017	2018	2019	2020	Total
Number of micropapillary cancers (males)	4	3	3	2	4	1	4	3	5	7	2	38
Number of papillary cancers (males)	19	12	14	18	27	19	18	25	35	39	25	251
Proportion of micropapillary carcinomas (males)	21.1	25.0	21.4	11.1	14.8	5.3	22.2	12.0	14.3	18.0	8.0	15.1
Cumulative numbers												
Year	2010	2011	2012	2013	2014	2015	2016	2017	2018	2019	2020	Total
Number of micropapillary cancers (males)	6	7	6	3	6	4	6	4	8	10	5	65
Number of papillary cancers (males)	31	22	28	34	47	31	28	35	45	49	33	383
Proportion of micropapillary carcinomas (males)	19.4	31.8	21.4	8.8	12.8	12.9	21.4	11.4	17.8	20.4	15.2	17.0

**Table 4.** Size distribution of PTCs.

Size (mm)	0–10	11–20	21–30	31–40	41–50	51–60	61–70	71–80	81–90	91–100	>100	No data
Thrissur Medical College	160	168	170	93	53	32	11	4	4	0	2	94
Calicut Medical College	181	236	211	113	54	25	14	11	5	4	4	25
Total	341	404	381	206	107	57	25	15	9	4	6	119
Proportion	20.3	24.1	22.8	12.3	6.4	3.4	1.5	1.0	0.6	0.2	0.4	7.1
